# Comparison of professionalism and job satisfaction between Korean midwives in birthing centers and midwives in hospitals

**DOI:** 10.4069/kjwhn.2020.09.08

**Published:** 2020-09-29

**Authors:** Buyoun Kim, Sook Jung Kang

**Affiliations:** College of Nursing, Ewha Womans University, Seoul, Korea

**Keywords:** Birthing centers, Hospitals, Job satisfaction, Midwifery, Professionalism

## Abstract

**Purpose:**

Midwives working in hospitals (MWH) have limited roles in managing and assisting births independently. To find ways to successfully integrate midwifery into care systems, exploring midwives’ work-related perceptions might be the first step. The purpose of this study was to compare professionalism and job satisfaction between Korean midwives working in birthing centers (MWBC) and MWH.

**Methods:**

A descriptive comparative design was used, querying 19 MWBC and 53 MWH in Korea. Data were accrued from October to November 2017 using the Professionalism Inventory Scale and the Job Satisfaction Scale.

**Results:**

Age, marital status, monthly income, length of career as a midwife, and length of career in the current workplace were significantly different between MWBC and MWH. The level of professionalism among MWBC showed significant differences by position at the birthing center (t=16.19, *p*=.001). Professionalism and job satisfaction among MWH showed significant differences depending on perceived professional performance (F=9.95, *p*<.001 and F=11.04, *p*<.001, respectively). Levels of professionalism and job satisfaction were higher for MWBC than for MWH.

**Conclusion:**

Educational programs designed to enhance professionalism and expand the role of MWH are suggested. Also, policy changes that clearly define job roles and improvement of the legal system is required, so MWH in Korea can effectively perform their midwifery work and be properly reimbursed.

## Introduction

The average number of midwives in 32 Organisation for Economic Co-operation and Development (OECD) countries in 2011 was 69.9 per 100,000 women [1], whereas Korea had the lowest, 3.4 midwives per 100,000 in 2015 [2]. Although Korea licensed about 20 midwives per year since 2011, midwives still constitute the smallest number among medical professionals [2]. Among the 8,266 licensed midwives in Korea in 2018, the number of midwives who actually work is less than 10% [2]. In Korea, midwives can work either in birthing centers or hospitals. According to 2015 data, the number of midwives working in hospitals (MWH) decreased steadily from 896 in 2007 to641, and only 39 midwives actively worked in 31 centers nationwide [2]. Without understanding the reason for this decrease, the number of midwives is expected to drop further.

The International Confederation of Midwives (ICM) defines the midwives’ scope of practice, emphasizing their full responsibility for supporting, caring, and advising mothers and their babies [3]. However this is not applicable to the current role and scope of practice among Korean midwives. That is, midwives in Korea are responsible for caring for women in labor independently only in birthing centers, but rarely in hospitals or other health care settings run by physicians [4]. Since the 1980s, an increase in hospital births led to a surge in the number of obstetricians and gynecologists in Korea. These trends changed midwives’ practices, restricting them in the use of medical devices (e.g., vacuum extractors and sonograms) and drug usage [5], leading to the shrinking of midwives’ scope of practice. In addition, these changes led to the closure of many birthing centers due to the decreased competitiveness of midwives in the Korean health care system [6]. Circumstances for midwives working in birthing centers (MWBC ) are also limiting, such as laws mandating a minimum number of birth cases for a program to be certified as an MWBC training program, or requiring reliance on technology with a medical team. These legal stipulations , however, do not reflect the current low birth rate and need to be reconsidered to increase the number of MWBC and extend the scope of practice of MWBC in Korea. Although 77.1% of midwives in Korea are employed in hospitals [2], virtually all practice as registered nurses, not as midwives [7,8].

One important characteristic of professionalism for midwives is autonomy [9]. MWBC take full responsibility to manage and lead the whole birth process. In contrast, MWH cannot easily manage and conduct births independently in Korea. Furthermore, in hospitals, some midwives are placed in a hierarchical relationship with obstetricians, relegated to the role of assisting the obstetrician [5]. Professionalism is a major factor influencing job satisfaction [10,11] and midwives’ job satisfaction is critical in developing their sense of professionalism [12].

Over the past two decades in Korea, the total fertility rate rapidly declined from 1.54 children per woman in 1997 to 0.98 children per woman in 2018, accompanied by a decrease in the sizes of birthing centers and numbers of midwives [2]. Midwife-led counseling and managing have been noted to improve confidence for giving birth and lead to positive birth experience [13,14]. For better health outcomes of mothers and newborns, certified midwives can play an important role in perinatal care.

There is a paucity of research about Korean midwives, however, with only one study exploring job satisfaction of MWH [15]. Considering the large number of MWH [2], and midwives who have distinct roles as MWBC, investigating the professionalism and job satisfaction of both MWBC and MWH is needed.

Thus, the purpose of this descriptive correlational study were (1) to investigate characteristics of MWBC and MWH, (2) to identify professionalism and job satisfaction according to characteristics of MWBC and MWH, (3) to compare professionalism and job satisfaction among MWBC and MWH, and (4) to identify the relationship between professionalism and job satisfaction.

## Methods

Ethics statement: This study was approved by the Institutional Review Board (IRB) of Ewha Womans University (EWIRB-18-3.0-20170901). Informed consent was obtained from participants.

### Study design

This descriptive correlational study investigated the relationship between professionalism and job satisfaction among MWBC and MWH in Korea.

### Participants

Definitions of midwives are as follows: MWBC are those who work alone or as part of a team and manage childbirth at a birthing center or home; MWH are those who work in the birthing room of a hospital. In the present study, inclusion criteria for MWBC were midwives who were certified and registered in the Korean Midwives Association (KMA) and currently work in a birthing center. Inclusion criteria for MWH were midwives who were certified and registered in the KMA and currently work in a hospital delivery room. There were a total of 22 MWBC nationwide and all were recruited through contact with birthing centers in Korea. A total of 72 midwives participated in this study, of which 19 were MWBC (two refused participation and one offered inadequate responses) and 53 were MWH. This number was sufficient for analysis estimated by G*power 3.1.9.2 for correlation analysis, with a middle to large effect size of 0.65 from Cohen’s criteria [16], a two-tailed error of 0.05, and 85% power.

### Measures

General characteristics included age, marital status, educational level, and monthly income; job characteristics included current workplace, length of career as a midwife, and length of career in their current workplace. In addition, MWBC were asked about their position in the birthing center and number of births they deliver monthly. MWH were asked about the size of the hospital (number of beds), and to assess perceived professional performance, were asked “What percentage of your professional ability as a midwife do you think you use in the delivery room?” Participants’ responses were divided into three stages; (1) less than 50%, (2) more than 50-79%, and (3)80% and greater.

#### Professionalism

Professionalism was measured using the Professionalism Inventory Scale developed by Hall [17], revised by Snizek [18], and translated into Korean by Baek and Kim-Godwin [19]. Permission to use the Korean version of the instrument was obtained. The scale has five subscales: use of professional organizations as a major reference (5 items), beliefs in public service (5 items), autonomy (5 items), belief in self-regulation (5 items), and sense of calling to the field (5 items). Participants were asked to rate each item on a Likert-type scale ranging from 1 (strongly disagree) to 5 (strongly agree). Higher scores indicated higher professionalism. Cronbach’s alpha for the original Korean version was .82 [19] and .83.

#### Job satisfaction

Job satisfaction was measured by the Attitude Scale to Measure Occupational Satisfaction of Hospital Nurses [20], translated into Korean [21] and revised to fit nurses in general hospitals [22]. Approval to use the instrument was obtained from the original developer. This 20-item scale includes seven subscales: pay (2 items), job prestige/status (3 items), midwife–doctor relationship (2 items), organizational requirements (3 items), autonomy (3 items), task requirements (4 items), and interactions with colleagues (3 items). The researchers revised and supplemented the questionnaire to be suitable for midwives. Some terminology was replaced to render the questionnaire specific to the working environment of midwives (e.g., midwives, physicians, health care coworkers, and birthing process). Content validity was verified twice: by five experts in the first round followed by three experts in the second round. By revising two items that had less than .8 on the content validity index, the final 20 questions applicable to midwives were identified. Participants rated each item on a Likert-scale from 1 (strongly disagree) to 5 (strongly agree). Higher scores indicated higher job satisfaction. Cronbach’s alpha for the Korean version [21] and revised version [22] were .77 and .77, respectively. In this study, Cronbach’s alpha was .78.

### Procedure

Data collection was done from October to November 2017 through direct visits, postal mailings, and mobile surveys. MWBC were targeted at all birthing centers registered with the KMA and 14 birthing centers nationwide in eight districts were contacted. In the case of MWH, the recruitment document was posted at one of the largest online communities for graduates of midwifery education institute. Data collection for MWH was also conducted in the delivery room of two general hospitals in Busan and Daegu. Participants received an informational leaflet about the study and voluntarily completed an informed consent form and a paper survey. For the online survey, participants could complete the questionnaire after clicking the informed consent button. All study participants received a small gift. A total of 72 midwives, including 19 MWBC (five direct visits, eight postal mailings, and six mobile surveys) and 53 MWH (17 postal mailings and 36 mobile surveys), participated in the study.

### Data analysis

Data were analyzed using IBM SPSS Statistics ver. 21.0 (IBM Corp., Armonk, NY, USA). General characteristics, professionalism, and job satisfaction were analyzed by frequency, percentage, mean, and standard deviation. Differences in professionalism and job satisfaction, according to general characteristics, were analyzed by t-test and analysis of variance; differences between professional and job satisfaction among MWBC and MWH were analyzed by t-test. To correct numerical differences for participants in both groups, the pooled-variance t-test and the Welch-Satterthwaite t-test were used. Pearson’s correlation coefficient was used to identify the relationship between professionalism and job satisfaction.

## RESULTS

### Characteristics of MWBC and MWH

Table 1 shows the general and job characteristics of MWBC and MWH. The mean length of career as a midwife (year) was two times higher for MWBC (25.26) than MWH (11.17), whereas the mean length of career in the current workplace (year) did not show a wide gap between MWBC and MWH (12.79 and 13.65, respectively). Sixteen MWBC (84.2%) who were owners of birthing centers and the number of births per month was 6.9. The mean of perceived professional performance for MWH was 60.2%.

### Differences in professionalism and job satisfaction according to midwives’ characteristics

Table 2 presents differences in professionalism and job satisfaction by characteristics among MWBC and MWH. The levels of professionalism according to characteristics among MWBC were significantly different, depending on marital status (F=5.98, *p*=.012) and position at the birthing center (t=16.19, *p*=.001). Professionalism scores of MWH decreased significantly, aligned with a decrease in perceived professional performance, categorized as 80% or more, 50% to 79%, and less than 50% (3.41, 3.34, and 3.00, respectively). Job satisfaction scores of MWH decreased significantly, with the decrease in perceived professional performance (3.57, 3.29, and 2.95, respectively).

### Differences in professionalism and job satisfaction among MWBC and MWH

Table 3 shows differences in levels of professionalism and job satisfaction between MWBC and MWH. The mean score of professionalism of MWBC was significantly higher than that of MWH (t=7.42, *p*<.001). All five subscales of professionalism showed significant differences. As far as the rank order of the subscales, the same results emerged in both groups, with the highest score for “a sense of calling to the field” and the lowest score for “use of the professional organization as a major reference.” The mean score of job satisfaction was also significantly higher for MWBC than MWH (t=7.42, *p*=.002).

### Correlations between professionalism and job satisfaction among MWBC and MWH

A significantly positive correlation was found between professionalism and job satisfaction in both MWBC (r=.49, *p*=.034) and MWH (r=.66, *p*<.001) groups.

## DISCUSSION

MWBC included midwives who run birthing centers and midwives who were employed. The professionalism score of MWBC who own and run birthing centers was significantly higher than the score of MWBC who were employed. More evidence needs to be accumulated, but managing and organizing a birthing center may make midwives feel a greater sense of responsibility and autonomy in their workplace. Identifying a specific scope of practice and exploring perceptions of those midwives in future studies may help in understanding this difference.

MWH in this study with perceived professional performance of less than 50% had the lowest professionalism score. In a previous study using the same scale, MWH who answered they perform to the best of their abilities with scores lower than 50%, scored lower on professionalism than registered nurses [23]. Special or higher positions at work as well as bigger hospital size appear to influence MWH’s sense of professionalism [11,24]. Also, a previous study showed that midwives working in women’s hospitals had a higher performance rate of maternity-related tasks than midwives working in general hospitals [8]. For MWH to have higher sense of professionalism at work, the type of hospital, job description at the unit, and current position appear to be important factors. In addition, in-depth study is needed to discern the factors limiting the role of MWH and to explore their perceptions of the workplace environment.

MWH scored significantly lower on professionalism and job satisfaction than MWBC. Some midwives are making efforts to establish their own roles by networking and self-development, such as participating in international conferences offered by the ICM, but only a few midwives attend and most of them are MWBC [25]. The KMA could enhance midwives’ sense of professionalism through regular membership meetings, seminars, or publication of a professional journal. Such activity could enhance the sense of professionalism of all midwives, providing midwives with current information and sharing issues of international midwifery.

Average scores on five professionalism subscales between MWBC and MWH were compared. MWH’s “sense of calling to the field” score (3.70 points) was significantly lower than that of MWBC (4.38 points), but had the highest score among other professionalism subscales. MWH in this study were working in delivery rooms, and even though their sense of calling score was lower than MWBC, they may still perceive that caring for a birthing woman is their calling. MWH’s autonomy score (3.16 points) was similar to that of general nurses (3.11 to 3.29 points) in a previous study that used the same instrument [11]. Midwives need to work autonomously to care for a woman who is delivering a baby. Standardized job descriptions for MWH need to be developed to ensure autonomy of their work.

The result of lower job-satisfaction level of MWH than their counterparts in birthing centers is similar to the results of a United Kingdom study of hospital midwives with lower job satisfaction than private midwives [26]. Another study in the Netherlands, however, showed that there was no difference in job satisfaction between MWBC and MWH [27]. Following the initiation of the national health insurance system in Korea in 1988, the trend of delivering babies in hospitals became the norm by the 1990s [5]. Throughout the last 30 years, little effort was made to change this trend. In 2017, the same year as this study, monthly births in Korea were merely 29,814 [2]; only about 0.02% of births occurred in birthing centers. It is unsurprising that the job satisfaction level of MWH was lower than MWBC because their role as midwives is not currently recognized in the obstetrician-led birth system in Korea. Lack of midwives’ autonomy under the obstetrician-centered structure in Korea may be one major reason MWH had low job satisfaction and a low sense of professionalism. MWH are placed in a work environment that does not recognize their role as a midwife, resulting in a vicious cycle in which their professionalism and job satisfaction decrease. In a midwife-led birthing system, however, midwives can practice under their own understanding of their role [14]. A detailed job description, including how to educate and assist pregnant women, how to manage the environment for childbirth, and responsibilities and rights of the midwife with guaranteed autonomy would empower not only MWBC but also MWH.

Previous studies were reported positive correlations between professionalism and job satisfaction among nurses [11,28]. Therefore, to enhance job satisfaction, perceptions of professionalism among midwives also need to be considered. Establishing midwifery at the graduate education level has long been suggested [4]; however, such a course of education is not yet available in Korea. All registered midwives are required to have 8 hours of continuing education per year [4], but this may be insufficient for midwives to keep abreast of knowledge and skills. Shin [4] suggested expanding continuing education to twice the current requirements, which would be 16 hours. Although this expansion may not be easy to develop at this time, it would enhance the quality and content of required education for midwives. Hands-on practical education needs to be included in current continuing education rather than online education or lecture-type education because on-the-job errors of midwives can have life-or-death consequences. In this sense, simulation education with a human-patient simulator, standardized patient, and virtual simulation may be conducive because those modes can enhance knowledge, skill practice, and communication skills.

The strength of this study is that it is one of the first comparative studies on professionalism and job satisfaction between MWBC and MWH in Korea. Despite a number of studies on women who use natural childbirth in birthing centers, studies on midwives have rarely been conducted. Another strength is the representativeness of the MWBC sample in this study, as all were contacted and the majority (19 out of 22) participated.

A limitation of this study is that the numbers of participants in the two groups were dissimilar. This was considered in statistical analysis, but remained a limitation of this study. Also, in the case of MWH, generalization is limited because of convenience sampling.

To improve the professionalism and job satisfaction of midwives, it is necessary to update continuing-education practice programs designed to enhance their sense of professionalism. National organizations such as the KMA should develop related practical educational programs. Also improving hospital policy can help clarify the role of MWH. Moreover, the legal system needs to include reimbursement so midwives are reasonably paid for their work. Lastly, Korean midwives need to rethink what changes can help them in placing the needs of mothers and their newborns at the center of the birthing process, aligned with the Lancet Midwifery Series [29].

## Figures and Tables

**Table 1.** Characteristics of MWBC and MWH (N=72)

Characteristic	Categories		n (%)	
			MWBC (n=19)	MWH (n=53)
Age (year)	20–29		0 (0)	8 (15.1)
	30–39		5 (26.3)	21 (39.6)
	40–49		1 (5.3)	20 (37.7)
	50–59		7 (36.8)	4 (7.5)
	≥ 60		6 (31.6)	0 (0.0)
Marital status	Married		16 (84.2)	29 (54.7)
	Single		2 (10.5)	23 (43.4)
	Divorced		1 (5.3)	1 (1.9)
Educational level	Diploma		9 (47.4)	13 (24.5)
	University		7 (36.8)	26 (49.1)
	Graduate school		3 (15.8)	14 (26.4)
Monthly income (10,000 KRW)	<300		6 (31.6)	25 (47.2)
	300–399		5 (26.3)	20 (37.7)
	400–499		3 (15.8)	6 (11.3)
	≥500		5 (26.3)	2 (3.8)
Length of career as midwife (year)		Mean±SD	25.26±11.97	11.17±6.54
	<10		2 (10.5)	20 (37.7)
	10–19		6 (31.6)	29 (54.7)
	20–29		3 (15.8)	4 (7.5)
	≥30		8 (42.1)	0 (0.0)
Length of career in current workplace (year)		Mean±SD	12.79±14.06	13.65±6.67
	<10		13 (68.4)	20 (37.7)
	10–19		2 (10.5)	29 (54.7)
	20–29		0 (0.0)	4 (7.5)
	≥30		4 (21.1)	0 (0)
Position at birthing center	Owner		16 (84.2)	
	Staff		3 (15.8)	
Number of births/month		Mean±SD	6.89±3.11	
Perceived professional performance (%)	<50			22 (41.5)
	50–79			23 (43.4)
	≥80			8 (15.1)

KRW: Korean won; MWBC: midwives working in birthing centers, MWH: midwives working in hospitals.

**Table 2.** Comparison of professionalism and job satisfaction by characteristics among participants (N=72)

Characteristic	Categories	MWBC (n=19)				MWH (n=53)			
		Professionalism		Job satisfaction		Professionalism		Job satisfaction	
		Mean±SD	t or F (*p*)	Mean±SD	t or F (*p*)	Mean±SD	t or F (*p*)	Mean±SD	t or F (*p*)
Age (year)	30–39	3.71±0.15	1.95 (.165)	3.53±0.16	0.36 (.783)	3.23±0.22	0.04 (.988)	3.15±0.44	0.12 (.951)
	40–49	3.44±0.00		3.25±0.00		3.19±0.40		3.22±0.49	
	50–59	3.96±0.08		3.56±0.37		3.22±0.35		3.16±0.31	
	≥60	3.91±0.09		3.51±0.25		3.22±0.21		3.25±0.39	
Marital status	Married	3.92±0.22	5.98 (.012)	3.55±0.28	1.16 (.337)	3.25±0.36	0.97 (.387)	3.23±0.37	2.26 (.115)
	Single	3.36±0.11		3.25±0.00		3.14±0.32		3.11±0.43	
	Divorced	3.76±0.00		3.60±0.00		3.48±0.00		3.90±0.00	
Educational level	Diploma	3.88±0.29	0.20 (.818)	3.53±0.27	0.03 (.970)	3.14±0.37	0.49 (.616)	3.16±0.32	0.05 (.950)
	University	3.80±0.29		3.50±0.23		3.25±0.29		3.20±0.40	
	Graduate school	3.90±0.22		3.53±0.46		3.20±0.41		3.20±0.51	
Monthly income (10,000 KRW)	<300	3.74±0.35	0.40 (.735)	3.40±0.23	1.05 (.399)	3.24±0.27	0.41 (.745)	3.13±0.32	0.72 (.542)
	300–399	3.90±0.27		3.55±0.35		3.20±0.41		3.19±0.49	
	400–499	3.91±0.22		3.73±0.12		3.21±0.35		3.41±0.46	
	≥500	3.90±0.24		3.51±0.28		2.96±0.51		3.20±0.35	
Length of career as midwife (year)	<10	3.84±0.11	1.77 (.196)	3.35±0.50	1.54 (.244)	3.23±0.28	0.61 (.549)	3.17±0.37	0.89 (.419)
	10–19	3.67±0.33		3.48±0.18		3.17±0.39		3.17±0.44	
	20–29	4.03±0.26		3.80±0.18		3.36±0.17		3.45±0.24	
	≥30	3.93±0.21		3.49±0.27		NA		NA	
Length of career in current workplace (year)	<10	3.84±0.23	0.08 (.921)	3.52±0.26	1.95 (.175)	3.23±0.27	0.17 (.845)	3.14±0.39	0.18 (.835)
	10–19	3.92±0.17		3.83±0.04		3.19±0.39		3.29±0.44	
	20–29	NA		NA		3.27±0.19		3.24±0.30	
	≥30	3.87±0.26		3.39±0.28		NA		NA	
Position at birthing center	Owner	3.93±0.21	16.19 (.001)	3.55±0.28	1.18 (.293)				
	Staff	3.43±0.14		3.37±0.20					
Perceived professional performance (%)	<50					3.00±0.31	9.95 (<.001)	2.95±0.40	11.04 (<.001)
	50–79					3.34±0.23		3.29±0.30	
	≥80					3.41±0.41		3.57±0.31	

KRW: Korean won; MWBC: midwives working in birthing centers; MWH: midwives working in hospitals; NA: not applicable (cell count reported 0).

**Table 3.** Comparison of professionalism and job satisfaction between the two midwife groups (N=72)

Variable	Subscales	Mean±SD		t	*p*
		MWBC (n=19)	MWH (n=53)		
Professionalism	Total	3.85±0.27	3.21±0.34	7.43	<.001
	Professional organization as reference	3.37±0.63	2.49±0.46	6.43	<.001
	Belief in public service	3.74±0.51	3.43±0.47	2.39	.020
	Autonomy	4.14±0.38	3.16±0.61	6.51	<.001
	Belief in self-regulation	3.64±0.49	3.27±0.61	2.40	.019
	Sense of calling to field	4.38±0.47	3.70±0.61	4.39	<.001
Job satisfaction	Total	3.52±0.28	3.19±0.41	3.98	.002
	Pay	3.29±1.02	2.42±0.79	3.80	<.001
	Job prestige/Status	4.44±0.59	3.84±0.76	3.10	.003
	Midwife–doctor relationship	2.95±0.76	3.47±0.85	–2.61	.011
	Organizational requirements	3.09±0.54	2.84±0.68	1.41	.164
	Autonomy	4.23±0.50	3.43±0.63	4.97	<.001
	Task requirements	2.76±0.54	2.33±0.45	3.38	.001
	Interaction with colleagues	3.88±0.72	4.10±0.55	–1.40	.167

All subscales were evaluated on a Likert-type scale from 1 (strongly disagree) to 5 (strongly agree). MWBC: midwives working in birthing centers; MWH: midwives working in hospitals.
